# Evaluation of an antenatal acupuncture intervention as an adjunct therapy for antenatal depression (AcuAnteDep): study protocol for a pragmatic randomised controlled trial

**DOI:** 10.1186/s13063-016-1204-9

**Published:** 2016-02-17

**Authors:** Simone M. Ormsby, Caroline A. Smith, Hannah G. Dahlen, Phillipa J. Hay, Joanne M. Lind

**Affiliations:** PhD Candidate, National Institute of Complementary Medicine, Western Sydney University, Building 5, Campbelltown Campus, Locked Bag 1797, Penrith, NSW 2751 Australia; Professor of Complementary Medicine, National Institute of Complementary Medicine, Western Sydney University, Building 5, Campbelltown Campus, Locked Bag 1797, Penrith, NSW 2751 Australia; Professor of Midwifery, Western Sydney University, Building EB, Parramatta Campus, Locked Bag 1797, Penrith, NSW 2751 Australia; Chair of Mental Health, Western Sydney University, Building 30, Campbelltown Campus, Locked Bag 1797, Penrith, NSW 2751 Australia; Associate Professor, Molecular Biology and Genetics, Western Sydney University, Building 30, Campbelltown Campus, Locked Bag 1797, Penrith, NSW 2751 Australia

**Keywords:** Acupuncture, Antenatal depression, Intervention, Pragmatic, Protocol, Randomised controlled trial

## Abstract

**Background:**

Depressed pregnant women face difficulty navigating a course between the potentially serious consequences of leaving depression untreated and significant limitations associated with conventional therapies, such as foetal toxicity and teratogenicity. Preliminary evidence is suggestive that acupuncture may provide a safe and effective alternative treatment option for antenatal depression; however, additional research is required. The purpose of this study is to further investigate this treatment possibility, with an additional examination of a potential biomechanistic acupuncture effect.

**Methods/design:**

In this pragmatic randomised controlled trial, we will compare individually tailored, flexible antenatal depression-oriented acupuncture with equivalent attention progressive muscle relaxation and routine antenatal depression hospital care. Eligible women at 24 weeks of gestation with Edinburgh Postnatal Depression Scale scores of 13 or more will be recruited from 2 antenatal clinics in South Western Sydney, Australia. The recruitment goal of 96 is powered to demonstrate a significant difference in Edinburgh Postnatal Depression Scale score severity between acupuncture and usual care, with intervention groups receiving weekly 1-h treatments for 8 weeks from 24 to 31 weeks of gestation. Mental health and quality-of-life assessments will occur at study commencement, intervention weeks 4 and 8 and 6 weeks post-natally via the collection of completed Edinburgh Postnatal Depression Scale scores, Depression, Stress and Anxiety Scale scores and World Health Organisation Quality of Life Scale scores. Adjustment to mothering will also be evaluated at 6 weeks post-natally using the Being a Mother Scale. A putative biomechanistic effect of acupuncture on the oxytocinergic system will additionally be examined by comparing baseline salivary hormone levels with those measured at intervention weeks 4 and 8, as well as leucocyte oxytocin receptor expression at baseline and intervention week 8.

**Discussion:**

Ethical approval was received in February 2015, and recruitment is underway and expected to be completed in July 2016.

**Trial registration:**

Australian New Zealand Clinical Trials Registry ACTRN12615000250538, Registered on 19 March 2015.

**Electronic supplementary material:**

The online version of this article (doi:10.1186/s13063-016-1204-9) contains supplementary material, which is available to authorized users.

## Background

In developed countries, including Australia, depression in pregnancy occurs at rates similar to those seen in non-pregnant women [1, 2], with prevalence reportedly ranging from 7.4 % [3] to 25 % [4] or higher [1] in vulnerable at risk groups [1, 4]. Whilst areas of lower socioeconomic status (SES) have not been identified as a risk factor in all studies [5], they have been shown to contribute to the presence of a greater number of individual psychosocial risks [6] and consequently may result in increased vulnerability. Morbid consequences include a significantly increased risk of obstetric complications [1, 4, 7–11] and post-natal depression in mothers [2, 12], as well as alterations in growth, development [11, 13], autonomic neuroendocrine function [4, 14–16] and mental health [4, 14, 17] in offspring. Treatment recommendations range from psychotherapy or anti-depressants for moderate cases to electroconvulsive therapy or a tricyclic combined with an anti-psychotic for depression with psychosis [18]. Prevailing medical opinion advocates that the consequences of leaving antenatal depression pharmacologically untreated is comparable to or greater than the risks associated with anti-depressant side effects and toxicity [11, 19, 20]. Yet, the efficacy of anti-depressants in pregnancy remains untested in large randomised controlled trials (RCTs), and, whilst expected to be comparable to those observed in non-pregnant populations [21], pregnancy-related pharmacokinetic changes may necessitate a need for increased dosage [20, 22]. Clinicians often reduce prescriptions in an attempt to limit foetal exposure, and resultant levels may therefore be subtherapeutic [22]. Recent evaluations of anti-depressant response rates in non-pregnant populations report modest improvements ranging from 14 % [23] to 32 % overall [24], and more than 50 % of cases failing to achieve remission [25]. Treatment compliance is also an issue, with 45 % partially [26] and 42 % completely discontinuing medication within the first 3 months [27, 28], due in part to unwanted adverse effects [27, 29]. This is especially the case in intentionally conceiving or pregnant women, in whom cessation of medication is particularly common [9, 29], resulting in relapse rates of 68 % compared to 26 % in those who continue medication [30]. Combined pharmacologic treatments and psychotherapies, although considered to be more efficacious, also appear to be limited in regard to depression severity and chronicity [31].

Studies indicate that depressed pregnant women are reluctant to take anti-depressant medication [9, 32] and are significantly more likely to voice a preference for non-pharmacologic options [33, 34]. Preliminary research into acupuncture for the management of antenatal depression, although limited to three clinical studies, appears promising and reflective of similar findings in systematic reviews of acupuncture as a treatment for depression [35–37]. In the first antenatal depression RCT, published in 2004, Manber and co-workers [38] compared 12 sessions of depression-specific, manualised acupuncture with ‘sham’ acupuncture and a massage control in 61 pregnant women with major depression. Significantly higher response rates were reported for depression-specific acupuncture (69 %) compared with massage (32 %) (*p* = 0.031) and a non-significant intermediate response rate in comparison to sham (47 %). The depression-specific acupuncture group also demonstrated a significantly higher average rate of reduction in depression scores within the first month of treatment compared with the massage control (*p* = 0.047). In 2010, Manber et al. [39] conducted a larger RCT of 150 pregnant women and demonstrated significantly decreased symptom severity in the acupuncture group (*p* < 0.05) compared with combined control subjects or sham alone, as well as significantly greater response rates (63.0 %) compared with combined controls (*p* < 0.05) or sham alone (*p* < 0.05). In 2007, Bosco Guerreiro da Silva [40] similarly investigated emotional complaints in pregnancy by quasi-randomising 51 women to a pre-programmed acupuncture protocol with four optional points or a non-treatment control. Symptom severity reportedly reduced by up to 50 % in 15 (60 %) of 25 subjects in the acupuncture group compared with 5 (26 %) of 19 in the control group (*p* = 0.013). In addition, significant reductions in the impact of distress in 3 of 5 life disturbance categories were also reported (*p* < 0.05). Whilst the findings of these three trials appear encouraging, the overall methodological quality is limited by small sample sizes [38, 40], quasi-randomisation and lack of an equivalent attention control [40], unclear randomisation generation and concealment [9, 38], unclear assessor blinding [9, 38, 40], and incomplete baseline and outcome data [9, 38]. Participant and practitioner blinding was performed in the two RCTs [38, 39], as was outcome assessor blinding in one [9, 39]. In all three studies, emotional distress was clinically evaluated.

How acupuncture may reduce depressive symptomology remains uncertain; however, one mechanism by which it may exert an effect is via the oxytocinergic system. This system, regulated by both oxytocin (OT) hormone levels and oxytocin receptor (OTR) activity [41], is responsive to stressors of both physical and psychological origin [42]. The resultant anti-anxiolytic, anti-depressive and stress reducing effects [41, 43] appear to result from adjustments to the hypothalamic–pituitary axis, autonomic nervous system activity [44] and reward centres in the brain [43]. A multitude of physical [42], psychosocial, emotional and behavioural functions [44–48], including mental health and parental-infant bonding [49], have been attributed to this system, and, not surprisingly, disruptions within it are also implicated in a plethora of negative emotional and social phenotypic traits [50]. Studies of the relationship between mental health and the oxytocinergic system are suggestive of dysregulation [51], as lowered [52–60] and elevated OT and OTR levels [51, 61–66], as well as dysregulated patterns of peripheral OT release [66], are seen in participants with depression in a variety of different observational and experimental conditions. The influence of acupuncture upon this system has also been assessed in 12 rodent studies and 1 human study, in which significant positive regulatory effects were reported, directly via radioimmunoassay and/or enzyme-immunoassay or quantitative real-time polymerase chain reaction (qRT-PCR) analysis [67–74], as well as indirectly via assessment of behaviour [75–79]. Further indirect evidence has been provided by 11 human studies examining acupuncture for the preparation, induction or enhancement of labour. Among these studies, significantly reduced requirements for syntocinin augmentation were reported in the acupuncture groups of nine studies [80–88], as was a trend toward less need in the acupuncture group of another [89]. In the remaining study, acupuncture was performed in addition to intravenous syntocinin, and labour effectiveness rates were also significantly improved [90]. Marginal non-significant elevations in syntocinin use were reported in two similar studies [91, 92]; however, methodological issues in both cases may have compromised the results.

As preliminary evidence is suggestive that acupuncture may be beneficial in reducing the severity of antenatal depression and may exert a regulatory effect upon the oxytocinergic system, additional research is warranted to determine whether acupuncture is a viable alternative therapeutic option. The aim of this study is to conduct an RCT to examine the hypotheses that acupuncture, compared with an attention comparator or usual care, reduces the severity and duration of antenatal depression, stress and anxiety; decreases adverse maternal and infant outcomes; improves maternal quality of life and infant bonding; and positively regulates OT and OTR expression.

## Methods/design

### Overview

A pragmatic, parallel-group RCT will be conducted to compare individually tailored, semi-standardised, depression-specific acupuncture with an equivalent attention progressive muscle relaxation (PMR) comparator and usual care. The morbidity of this population is sufficiently serious to maintain existing treatment and evaluate the interventions as adjunctive therapies [93]. The trial design incorporates individualised treatment flexibility [94], typical of a ‘whole systems’ approach [95, 96], within the framework of a semi-standardised protocol [97–100] and is thereby reflective of the recent emphasis on effectiveness studies of acupuncture interventions to maintain ecological validity [101] as well as generalisability and interpretability [98, 99]. The non-specific, placebo-like effects resulting from intervention participation [102, 103] will be estimated via the attention comparator, whereas antenatal depression progression or remission and compliance to standard therapy will be monitored via the non-treatment control [100].

Ethical approval for this study was granted in February 2015 by the Research and Ethics Office of the New South Wales Department of Health, South West Sydney Local Health District (SWSLHD-HREC/14/LPOOL/400) and the Western Sydney University Human Research Ethics Committee (WSU-HREC/H10993). The trial is registered with the Australian New Zealand Clinical Trials Registry (ACTRN12615000250538). The protocol (version 1.0) has been designed in accordance with the SPIRIT (Standard Protocol Items: Recommendations for Interventional Trials) guidelines for interventional trials [104, 105] (see Additional file 1) and will be conducted in accordance with the Declaration of Helsinki (1964) and the International Conference on Harmonization Good Clinical Practice (1996). Any changes that need to be made to the trial protocol will be communicated to all investigators, the ethics committees and the trial registry.

### Eligibility criteria

Women will be eligible if they are at 24 weeks of gestation, ≥18 years of age, have mood disorders and score ≥13 on the Edinburgh Postnatal Depression Scale (EPDS) [106] (indicative of a high probability of current depression) [106, 107]. Women will be excluded if they are experiencing a medically diagnosed major depressive episode ≥2 years in duration and/or have psychotic or manic features rendering them incapable of consent, post-traumatic stress disorder with needle phobia, a current psychiatric assessment of suicidal risk, a condition necessitating bed rest, and/or other major obstetric risks. Participants must also agree not to receive acupuncture or PMR other than that provided within the study.

### Recruitment, setting and informed consent

The study is being conducted at two hospital sites in South Western Sydney, Australia. Both sites provide antenatal outpatient services and are under the governance of the same health authority; however, birthing services are available only in the larger of the two. The region serviced includes suburbs considered to be of disadvantaged SES [108], high ethnic diversity and greater than state average indigenous population density [108, 109]. Pregnant women will be introduced to the study via flyers included in antenatal information packs. Antenatal staff will additionally supply a study poster to all women identified with elevated EPDS scores during routine antenatal screening. Details of these women will be provided to the researcher, who, upon contact, will forward participant information sheets to all interested potential recruits. Once agreeing to join the study, all women will be further screened and, if eligible, randomised after signed informed consent forms containing separate provisions for the collection and use of biological specimens have been obtained in every case (Additional file 2).

### Randomisation and blinding

Participants and practitioners are not blinded to the study; however, data entry and analysis will be blinded. The randomisation schedule was computer-generated to contain three groups of random numbers, enabling stratification for the three different models of antenatal care (obstetrician, standard and caseload midwifery) as well as to randomly contain ‘blocks of three’ within each group, acupuncture, PMR or usual antenatal hospital care (1:1:1). The schedule was generated by the school statistician (Paul Fahey) and concealed in opaque envelopes by an independent researcher from the National Institute of Complementary Medicine (NICM) (Anthony Good).

### Treatment and assessment schedules

**Fig. 1:**
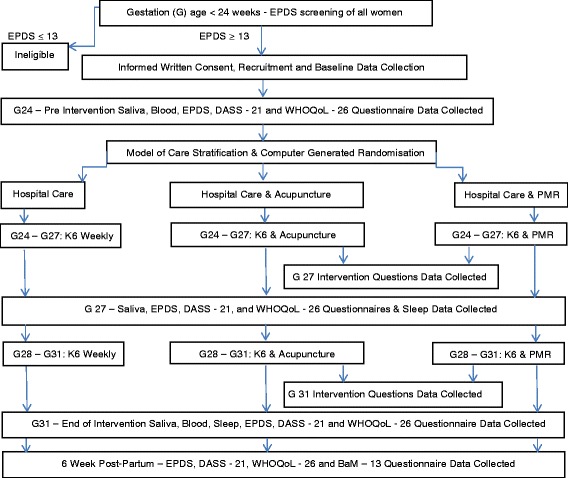
Pragmatic randomised controlled trial treatment flowchart. *EPDS* Edinburgh Postnatal Depression Scale, *G* gestational week, *DASS-21* 21-item Depression Anxiety and Stress Scale, *WHO-QoL-26* 26-item World Health Organisation Quality of Life Scale, *K6* Kessler 6 instrument, *PMR* progressive muscle relaxation, *BaM-13* 13-item Being a Mother Scale

The study period will run for 8 consecutive weeks from gestation week 24 to the end of week 31 (Fig. 1). At baseline, age, gestational age, number of previous pregnancies and/or births, relationship status and/or quality, education, employment status and country of birth will be collected, along with depression-related clinical data, including medical diagnoses, entry EPDS score, age of onset and length of index episode, number and severity of past depressive episodes, length and severity of current episode, family history of depression and current medication and/or psychotherapy use. Collected data will be de-identified and securely stored at the NICM.

### Safety monitoring

Owing to the morbidity of this population, women will undergo a weekly psychological assessment with the Kessler 6 (K6) instrument [110], either during the weekly session (intervention groups) or by telephone (usual care). If notable worsening or a ‘cut-off’ score is reached, immediate referral will be made to hospital antenatal mental health services. Any adverse events arising from the acupuncture intervention will be documented and reported to Western Sydney University and the South West Sydney Local Health District, and prevalence will be calculated. Such events are reportedly rare (occurring approximately 1.3 times every 1000 treatments) and generally of a mild [111, 112] to moderate nature [112]. However, if such events do occur, participants will be reminded of their right to withdraw from the study at any time.

### Interventions

In the first session, a full case history will be taken and a traditional Chinese medicine (TCM) and meridian therapy diagnosis will be made. This will also occur for the usual care group upon trial entry. The intervention groups will then receive weekly 1-h treatments over the 8-week period. At the end of each session, 5 minutes will be spent gaining feedback regarding the session. Treatments will be conducted in the antenatal clinics of either hospital or at the NICM.

**Fig. 2:**
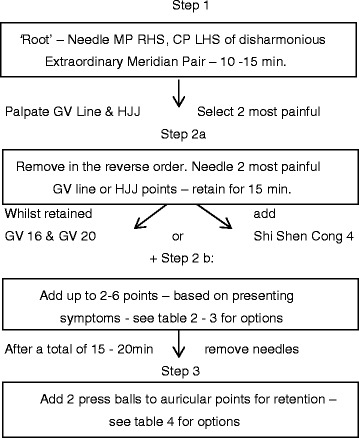
Acupuncture treatment flowchart. *CP* coupled points, *GV* governor vessel, *HJJ* Huato Jia Ji, *LHS* left-hand side, *MP* master points, *RHS* right-hand side

The acupuncture protocol draws upon TCM theoretical foundations and treatment strategies as well as some generalisable traditional East Asian medicine approaches. The three-stage semi-standardised flexible process (Fig. 2) incorporates a ‘root’- and ‘branch’-style treatment [113, 114] as well as auricular acupuncture. The ‘root’ treatment (step 1) aims to provide a fundamental harmonisation via the extraordinary meridian system, while the ‘branch’ treatment (step 2) focuses on remaining disruptions in a more symptomatic way [114–116]. Use of the extraordinary meridian system as a root-style approach has been suggested to facilitate a longer-lasting, more penetrating effect [114, 116] by virtue of this system’s ability to regulate the ‘qi’ of all of the yin-and-yang channels [117], access and distribute ‘pre-heaven essence’ throughout the entire body [116, 118] and affect a person at a constitutional level, due to its embryological roots [116, 118, 119]. Specific functions relevant to depression include influences to the marrow [116], brain, spinal chord, hypothalamic–pituitary–ovarian axis [120], circulatory, hepatic, biliary [121] and endocrine [118] systems. Pathology within this system is thought to manifest in the mind or ‘shen’ [118], and as such treatment is recommended for mental and/or emotional problems [118, 120] as well as cases of mixed pattern complexity involving multiple meridians [118]. In step 1, diagnosis of the most disharmonious extraordinary meridian pair will be made on the basis of the presence of relevant mood disturbance symptomology [116] in combination with disease indicating palpatory findings along involved channel trajectories [117–119]. If uncertainty remains regarding which of the 2 points of the meridian pair will be the master point (MP), each point will be tested with acupressure to see which one provides the greatest meridian improvement. After identification, the gender-appropriate MP and coupled point (CP) (Table 1) will be unilaterally needled with the meridian flow to ensure that the areas traversed by both vessels are influenced [118]. This method was recommended by Maciocia in 2006 [118] for problems of the head and internal organs, weakened body condition and anxiety. It is also suitable for pregnant women in side-lying position. Japanese-style practitioners typically apply polarity devices to the MP and CP to remedy detected pathologies in the extraordinary vessel system [119, 122]. However, for the purposes of generalisability, polarity in this case will be achieved by side of sex-directed unilateral needling and reverse-order withdrawal [116]. Whilst needles are retained for 10–15 minutes, further diagnosis will be performed by palpation and questioning. As many of the symptoms of depression correspond to TCM ‘yang deficiency’ such as lethargy, reduced libido and lack of motivation [116], it has been theorised that disruption to the major yang channel, the extraordinary meridian governor vessel (GV) that traverses the middle of the back and head, is implicated in depression [123]. Treatment recommended by Wang and Zhang in 2010 [123] is the needling of the most painful or obstructed points along this channel or on the slightly adjacent Huato Jia Ji (HJJ) points, serving a dual main channel and extraordinary vessel function [118]. Step 2a will involve needling of the two points located in this way, in combination with either GV 20 (bai hui) [124] + GV 16 (feng fu) [124], the ‘Sea of Marrow’, indicated for mania, suicidal tendencies and calming the spirit; or ‘Shi Shen Cong 4-4 Alert spirits’, indicated for mania, depression, insomnia and calming of the spirit [117]. Selection in either case will be based upon palpation tenderness and/or symptom differentiation. Whilst these needles are retained, step 2b will involve the needling of two to six additional tender points, if required, according to numerous theoretical possibilities (see Tables 2 and 3) such as the ability to clear reflex areas, for a combined total time of 15–20 minutes. A step 2b example is the association between depression and inflammation [125] viewed as ‘heat’ in TCM; hence, if detected at a ‘fire’ point on a meridian trajectory, a remedying treatment involving the needling of ‘water’ and ‘metal’ points’ [116] to extinguish the ‘fire’, may be selected. Step 3 will then comprise two stainless steel pellets (Helio Supply Co., San Jose, CA, USA) being placed on appropriate points in the ear (Table 4) to extend the treatment effect [122] for an additional 5 days. Lifestyle and dietary advice, contraindicated labour augmentation points [126] and painful needling techniques will be avoided [127]. Needles will be inserted into the most sensitive area within the specified point location or ‘live’ point [100] to a depth enabling retention (2–6 mm), either perpendicularly (even technique) or obliquely with the meridian flow (tonification) and taped in place to allow for adjustment of position during the session. AcuGlide (Helio Supply Co.) single-use stainless steel needles 0.16 mm × 30 mm will be used predominantly, with 0.14-mm or 0.12-mm needles selected for tenderer locations.

**Table 1:** Master and coupled point pairings of the eight extra vessels

Extraordinary meridian	Master point (right)	Couple point (left)
Ren Mai – Directing Vessel	Lung (LU) 7 (lie que)^a^	Kidney (KD) 6
Yin Qiao Mai –Yin Heel Vessel	Kidney (KD) 6 (zhao hai)	Lung (LU) 7
Chong Mai – Penetrating Vessel	Spleen (SP) 4 (gong sun)	Pericardium (PC) 6
Yin Wei Mai – Yin Linking Vessel	Pericardium (PC) 6 (nei guan)	Spleen (SP) 4
Dai Mai – Girdle Vessel	Gallbladder (GB) 41(zu lin qi)	Triple burner (TB) 5
Yang Wei Mai – Yang Linking Vessel	Triple burner (TB) 5 (wai guan)	Gallbladder (GB) 41
Du Mai – Governing Vessel	Small intestine (SI) 3 (hou xi)	Bladder (BL) 62
Yang Qiao Mai – Yang Heel Vessel	Bladder (BL) 62 (shen mai)	Small intestine (SI) 3

The eight extra vessels are described by Maciocia [116].

^a^From Ellis et al. [124]

**Table 2:** Step 3: Additional relevant point possibilities

Symptom and theoretical foundation	Points
Severe Yang deficiency^a^	GV 4 (ming men)^b^ and GV 14 (da zhui) or GV 4 and TB 4 (yang chi)
Anxiety + pressure pain at PC 8 (lao gong) or SP 2 (da du) – pericardium or spleen metal and water points^c,d^	PC 3 (qu ze) and 5 (jian shu) or SP 5 (shang qiu) and 9 (yin ling quan)
Anxiety + pressure pain at CV 12 (zhong wan) – points of the heart shu area^d^	T5/HJJ/BL 15 (xin shu)/BL 44 (shen tang)/BL 49 (yi she)
Anxiety + pressure pain at CV 17 (dan zhong)^d^	GB 13 (ben shen)
Depression/mental disturbance – ‘blood stagnation in the head’^c^	First – SP 9 (yu ling quan) second – PC 8 or PC 6 or PC 3 + 5
Depression due to adrenal exhaustion^c,d^	KD 6 and lateral to LU 5 (chi ze) or KD 6 and KD 27 (shu fu)
Depression and sphenoid bone disturbance – pressure pain at GB 4 (han yan)/5 (xuan lu)/6 (xuan li)^d^	Needle most tender
Depression and sphenoid bone disturbance – pressure pain TB 17 (yi feng) and TMJ^c^	KD 9 (zhu bin) and 27 and above TB 9 (si du) side of pain
Depression and pituitary disturbance^c^	SI 3, SI 13 (qu yuan), BL 2 (zan zhu) and 1 cun above BL 10 (tian zhu)
Melancholic depression^d^ – if positive for pressure pain/pulsation at CV 9 (shui fen)^d^	ST 24 (hua rou men) RHS, LU 9 (tai yuan)
Depression – LR 2 (xing jian) pressure pain – water and metal points^c^	LR 4 (zhong feng) and 8 (qu quan)
Depression – pressure pain LR organ region and LR 14 (qi men)^c^	LHS – lateral to LU 5 (chi ze), LR 4 and LR 14 RHS
Chronic depression and weakened immune system – pressure pain R ST 26 (wai ling)/27 (da ju ) and TB 16 (tian you)^c^	Tender point around LI 10 (shou san li)/11 (qu chi)
Subclinical underactive thyroid gland^c^	Eustachian, TB 4, KD 9/10 (yin gu), BL 43 (gao huang shu), HJJ C6/7
Inflammation – TCM meridian heat signs – metal and water points to extinguish^e^	Meridian-dependent
Mental health relevant points – selected by point function and palpation tenderness	See Table 3 for options

*BL* bladder meridian, *CV* conception vessel, *GB* gallbladder meridian, *GV* governor vessel, *HJJ* Huato Jia Ji, *KD* kidney meridian, *LHS* left-hand side, *LU* lung meridian, *LI* large intestine meridian, *LR* liver meridian, *PC* pericardium meridian, *SP* spleen meridian, *ST* stomach meridian, *RHS* right-hand side, *SI* small intestine meridian, *TB* triple burner meridian, *TCM* traditional Chinese medicine

^a^Maciocia [116]

^b^Ellis et al. [124]

^c^Matsumoto and Euler [134]

^d^Matsumoto and Euler [135]

^e^Kendall-Tackett [125]

**Table 3:** Possible points and point combinations useful for the treatment of antenatal depression

Points	Indications/Symptom alleviation
ST 40 (feng long)^a^	Spleen deficiency, damp and phlegm misting – heaviness, confusion, metal disturbance
ST 23 (tai yi)	Transforms phlegm and calms the spirit – mania, depression, agitation
ST 24 (hua rou men)	Transforms phlegm and calms the spirit – mania, depression
LR 3 (tai chong)	LR Qi stagnation, irritability, feeling of oppression
BL 42(po hu)	Breathing difficulties, grief, worry, sadness
BL 43	Increases spleen yang and lung function, calms the spirit, resolves phlegm
BL 44	Calming and mental clarity
BL 47 (hun men)	Enables clear and positive life planning and decision making
BL 47 and BL 52(zhi shi)	Together adds stability to plans and decisions, also for LR Qi stasis attack on lungs
BL 49	Obsessive thinking, improved memory and concentration
BL 52	Willpower, determination, initiation
BL 52 and BL 42	Willpower and emotional release
BL 52 and BL 44	Willpower, calming, anxiety, depression, mental restlessness and insomnia
BL 52 and BL 47	Willpower and life direction, chronic depression with mental exhaustion, apathy and despondency
BL 52 and BL 49	Willpower, obsessive thoughts, worry and confusion
BL 10 (tian zhu)	Calms the spirit
BL 17 (ge shu)	Mania, depression
GV 18 (qiang jian)	Liver blood deficiency, calming, mental restlessness, agitation, confusion, obsessive thinking
GV 19(hou ding)	Willpower, calming, anxiety, mental restlessness, kidney yin deficiency heat
GV 19 and CV 15 (jiu wei)	Calming, insomnia, mental restlessness
GV 20 (bai hui)	Lifts mood, aids memory and concentration
GV 21(qian ding)	Anxiety, insomnia, depression
GV 24 (shen ting)	Clears the mind
GV 24 and GB 13	Calming, anxiety, mental restlessness due to LR causes
CV 14 (ju que)	Transforms phlegm and calms the spirit
CV 15	Calming, chest oppression
GB 9 (tian chong)	Calms the spirit and pacifies fright
GB 12(wang gu)	Mania, agitation of the heart and insomnia
GB 13	Calming, mental clarity, severe anxiety and mental restlessness resulting from LR disharmony
GB 13 and GV 24	Same as for GB 13 with enhanced calming effect
GB 13 and HT 7 (shen men)	Calming, severe anxiety due to heart disharmony
GB 15 (tou lin qi)	Calms the spirit, reduces emotional fluctuations and obsessive thinking
GB 17 (zheng ying)	Calms the spirit, aids memory and concentration
GB 18 (cheng ling)	Stops obsessive thoughts, strengthens the function of the lungs and clears the nose
GB 40 (qiu xu)	Willpower, difficulty making decisions
HT 7	Calms the spirit, heart blood and yin deficiency
HT 6 (tong li)	Calms the spirit, repletion patterns with vacuity heat
HT 8 (shao fu)	Calms the spirit – agitation, fright palpitations, depleted sadness and worry
HT 9 (shao chong)	Calms the spirit – mania, depression, severe mental restlessness, anxiety, insomnia
HT 5 (tong li)	Calms the spirit – depressive disorder
PC 4 (xi men)	Calms the spirit – agitation, insomnia, melancholy, spirit qi deficiency
HT 7 and PC 6	Calms the spirit – shen disturbance, anxiety, mental restlessness
KD 7 ( fu liu) and HT 6 (yin xi)	Calms the spirit – heart and kidney not communicating, agitation, insomnia, anxiety
PC 5	Settles and calms the spirit – mania and agitation
PC 7 (da ling)	Calms the spirit – depressive disorder
PC 6	Calms the spirit, lifts mood – qi stagnation emotional problems, anger, resentment and frustration
PC 6 and SP 4	Opens yin wei mai, nourishes blood, relaxes the chest, calms the spirit
PC 6 and LR 3	Resolves qi stasis from repressed anger
PC 6 and GV 20	Lifts the mood, relieves depression
PC 6 and GV 26 (shu gou)	Lifts the mood, opens heart orifices, relieves depression
SI 7 (zhi zheng)	Calms the spirit – mania, depression, anxiety, sadness, fear and fright
KD 9 (zhu bin)	Clears the heart and transforms phlegm – mental restlessness, mania, depressive disorders
SI 7 and HT 3 (shao hai)	Chronic anxiety, depression, fear or emotional distress
LI 5 (yang xi)	Calms the spirit – mania, depression

*BL* bladder meridian, *CV* conception vessel, *GB* gallbladder meridian, *GV* governor vessel, *HJJ* Huato Jia Ji, *KD* kidney meridian, *LHS* left-hand side, *LU* lung meridian, *LI* large intestine meridian, *LR* liver meridian, *PC* pericardium meridian, *SP* spleen meridian, *ST* stomach meridian, *RHS* right-hand side, *SI* small intestine meridian

Information is from MacPherson and Schroer [98]; Schnyer and Allen [99]; Deadman et al. [117]; Matsumoto and Euler [136]; and Rogers and Rogers [147]

^a^Ellis et al. [124]

**Table 4:** Auricular point possibilities

Name	Function
Point Zero	Homeostasis, will power
Shen men	Pain, tension, anxiety and depression
Sympathetic tone	Sympathetic nervous system balance
Cheerfulness	Relieves depression
Excitement	Relieves hypersomnia and depression
Master cerebral point	Anxiety, worry, obsessive compulsive disorders, psychosomatic disorders and chronic pain
Master oscillation point	Balances left and right cerebral hemispheres
Sleep disorders 1	Relieves insomnia, nervousness
Sleep disorders 2	Relieves insomnia, sleep difficulties, nervous dreams
Depression	Relieves depression
Endocrine	Hormone imbalance
Nervousness	Relieves nervousness
Adrenal	Adrenal fatigue/disturbance
Thyroid 1–4	Thyroid function disturbance
Marvellous/wonderful	Lifts mood
Aggression	Alleviates aggression/irritability
Mania	Relieves manic symptomology
Insomnia	For sleep disturbance
Vitality	For lethargy and despondency
Stress control	Relieves stress
Tranquilizer	Calms the spirit
Guilt	Alleviates feeling of guilt
Overwhelmed	Stress, emotional paralysis, despondency
Burdened	Emotional paralysis, despondency, overwhelm

Adapted from several different Chinese medicine–based auricular maps obtained from the internet: https://www.google.com.au/search?q=chinese+auricular+ear+acupuncture+chart&biw=1140&bih=610&tbm=isch&tbo=u&source=univ&sa=X&ved=0ahUKEwjO6ofuuebKAhXEUZQKHR-UAAkQsAQIGg.

In our examination of recent reviews of acupuncture as a treatment for depression [35, 36, 128–130], we identified three main treatment approaches previously used: (1) fixed points for mood disturbance alleviation, (2) fixed points of this function in combination with additional points selected according to TCM pattern differentiation and (3) flexible point selection based entirely upon TCM pattern discrimination. Variations included diagnosis of disrupted extraordinary vessels Chong and Ren in one study [131], an abdominal points focus in another [132] and scalp acupuncture in a third [133]. Many studies included auricular acupuncture as well as multiple points along the extraordinary GV meridian. Two research groups developed flexible manualised protocols based on pattern differentiation [98, 99], one of which also provided modifications for pregnancy [99]. Both have been drawn upon for therapeutic possibilities presented in Tables 2 and 3, in combination with those suggested by Matsumoto and Euler in 2002 [134] and 2008 [135], such as for the alleviation of commonly observed LR Qi stagnation [98]. It is theorised that the approach of extraordinary meridian treatment in combination with mental disturbance–oriented pattern differentiation and symptom alleviation may provide additional therapeutic outcomes. In 2009, Finston [136] used a similar strategy in patients with severe mental disorders and reported significant reduction or alleviation of psychotic symptoms [136].

The PMR comparator has been adapted from previous research [137] to achieve overall body relaxation in all sessions with an additional weekly focus as follows: introduction (week 1); lower legs and knees (week 2); upper legs and buttocks (week 3); lower back and pelvic floor (week 4); upper back and chest (week 5); arms and shoulders (week 6); head, face and neck (week 7); and integration (week 8). Participants, whilst lying in a comfortable position, will be guided through the PMR in a one-to-one format. A participant who is unable to attend will be offered either a recording- or telephone-based session for home administration.

All groups will continue standard antenatal care, which may include medication and or counselling as well as antenatal classes. In compensation for not receiving treatment, the usual care group will be offered four acupuncture sessions after collection of final data at 6 weeks post-natally.

The principal investigator of this trial (SMO) has 14 years of full-time clinical experience and will conduct all acupuncture sessions, unless unable, in which case a backup acupuncturist with 14 years of part-time clinical experience will be employed. Both practitioners have bachelor’s degrees in acupuncture and are registered with the Chinese Medicine Board of Australia and the Australian Acupuncture and Chinese Medicine Association. The principal investigator will also conduct all PMR sessions, unless unable, in which case a backup associate researcher will be employed.

### Outcome measures

Data and samples will be collected at various time points, including baseline, mid-intervention (week 4), end of intervention (week 8), birthing and hospital discharge (medical records) and 6 weeks post-natally. All questionnaires will be self-administered and collected by the principal investigator. The primary outcome endpoint, reduction in depression severity, will be assessed at baseline, at 4 and 8 weeks from trial entry and at 6 weeks post-natally via the EPDS, an extensively validated, clinically sensitive [138], 10-item self-report questionnaire. Additional secondary outcome endpoints will also be examined as follows :

1. At the same time points as the EPDS, reduction in stress and anxiety will be monitored with the 21-item Depression Anxiety and Stress Scale (DASS-21) [139] and improvement to quality of life will be evaluated using the 26-item World Health Organisation Quality of Life Scale (WHO-QoL-26,) [140]. The DASS-21 is a short form of the clinically validated [141] and cross-culturally reliable [142] self-report measure of emotional states of stress and anxiety. The WHO-QoL-26 [140] is also an abbreviated form of this discriminant, content valid, internally consistent and reliable self-report measure for overall quality of life.
2. Adjustment to parenting and maternal-infant bonding will be evaluated at 6 weeks post-natally via the 13-item Being a Mother Scale (BaM-13) [143]. The BaM-13 is a clinically discriminating, reliable and valid self-report tool specifically designed to enable early assessment of women’s experiences of motherhood.
3. Maternal-infant outcomes such as gestational diabetes, mode of delivery, method of pain relief, labour augmentation, post-partum haemorrhage, breastfeeding level and duration, gestational age at delivery, birth weight, 1- and 5-minute Apgar scores, neonatal intensive care unit admission and need and reason for resuscitation and or intensive care will be assessed at delivery, discharge and 6 weeks post-natally.
4. Salivary OT levels will be determined using saliva specimens collected by the principal investigator at baseline and at 4 and 8 weeks from trial entry. Leucocyte OTR messenger RNA (mRNA) expression will be assessed using blood collected by phlebotomists at local pathology centres at baseline and 8 weeks from trial entry.

Every effort will be made to minimise missing data by ensuring weekly contact with participants during the intervention period as well as robust follow-up at later data collection points. Missing data for the primary endpoint of depression will be estimated using multiple imputation data derived from the K6 instrument. All missing data will be reported with reasons given and patterns of occurrence explored.

### Sample analysis

The principal investigator will process samples as follows. 4 ml of saliva will be obtained via passive drool into sterile CELLSTAR polypropylene test tubes (15 ml, catalogue number 188271; Greiner Bio-One, Frickenhausen, Germany), transported on ice and frozen and stored at −20 °C. Thawed samples will later be batch-processed and concentrated using equilibrated Sep-Pak C18 200-mg resin cartridges (catalogue number WAT 054945; Waters Corporation, Milford, MA, USA) before refreezing at −20 °C in 5-ml sterile Eppendorf tubes (catalogue number 0030119401, Eppendorf, Hauppauge, NY, USA). Triplicate OT concentrations will be batch-calculated using an OT enzyme-linked immunosorbent assay (ELISA) kit (catalogue number ADI-901-153A-0001; Enzo Life Sciences, Farmingdale, NY, USA) and an ELISA plate reader. Blood (2.5 ml) collected into PAXgene blood tubes (catalogue number 762165; BD Diagnostics, Hunt Valley, MD, USA) will be stored at −20 °C no sooner than 2 h after collection. Batch extraction of mRNA will later be performed using PAXgene blood RNA kits (catalogue number 762174; BD Diagnostics) and stored at −20 °C before cDNA libraries are made using iScript Advanced cDNA Synthesis kits (170-8843 Bio-Rad Laboratories, Hercules, CA, USA) and mRNA expression is determined by qRT-PCR using human OTR PCR primers (catalogue number VHPS-6555; Applied Biosystems, Foster City, CA, USA).

### Sample size

Mean antenatal EPDS scores extracted from a recent Australian study [111], as well as estimated post-intervention between-groups differences derived from a meta-analysis of two antenatal depression acupuncture studies [9], were used for a power calculation for this study. Using a power of 80 % and two-sided testing at a 5 % significance level, detecting a significant difference in end of intervention mean ± standard deviation (SD) EPDS scores of 8.9 ± 5 for the acupuncture group plus usual hospital care versus 13 ± 6.5 for usual hospital care will require a total recruitment goal of 75 participants. As high attrition rates have been reported for this population [144], an additional recruitment of 30 % has been added, resulting in a total goal of 96, or 32 participants per group.

### Statistical analysis

Descriptive statistics will be used to describe the characteristics of the study population. An intention-to-treat analysis will be undertaken [145], with between-groups differences explored using analysis of variance for the primary and secondary endpoints of depression, stress, anxiety, quality of life and OT/OTR. Effect sizes will be reported with 95 % confidence intervals, and results will be considered significant if *p* values are less than 0.05.

## Discussion

In consideration of the sufficiently serious consequences of antenatal depression in combination with therapeutic deficiencies, a thorough evaluation of any promising preventative and or therapeutic techniques that may be effective in reducing antenatal distress is required. As to date no studies have been conducted to investigate an acupuncture protocol of this type in depressed pregnant women or to evaluate the effects of acupuncture on the oxytocinergic system in this regard, it is the aim of this RCT to collect such data as well as to assess the usefulness of this acupuncture treatment for the alleviation of antenatal depression. Limitations of this study include the current lack of research evidence regarding the effectiveness of the proposed acupuncture treatment strategy, the use of self-report measures for the determination of treatment effect, a ‘practice effect’ that may occur due to repeated use of self-report measures and the unquantifiable possible effects of PMR that are in addition to non-specific placebo effects resulting from intervention procedures and interactions.

### Trial status

Recruitment is ongoing.

## Additional files

Additional file 1:
**SPIRIT 2013 Checklist.** (DOC 124 kb) Additional file 2:
**Consent form.** (PDF 204 kb)

## Abbreviations

BaM-13: 13-item Being a Mother Scale
BL: bladder meridian
CP: coupled points
CV: conception vessel
DASS-21: 21-item Depression Anxiety and Stress Scale
ELISA: enzyme-linked immunosorbent assay
EPDS: Edinburgh Postnatal Depression Scale
GB: gallbladder meridian
GV: governor vessel
HJJ: Huato Jia Ji
HT: heart meridian
K6: Kessler 6
KD: kidney meridian
LHS: left-hand side
LU: lung meridian
LI: large intestine meridian
LR: liver meridian
MP: master point
mRNA: messenger RNA
NICM: National Institute of Complementary Medicine
OT: oxytocin
OTR: oxytocin receptor
PC: pericardium meridian
PMR: progressive muscle relaxation
qRT-PCR: quantitative real-time polymerase chain reaction
SD: standard deviation
SP: spleen meridian
ST: stomach meridian
RCT: randomised controlled trial
RHS: right-hand side
SES: socioeconomic status
SI: small intestine meridian
TB: triple burner meridian
TCM: traditional Chinese medicine
TMJ: temporomandibular joint
WHO-QoL-26: 26-item World Health Organisation Quality of Life Scale
