# Supplementary material for: Evaluation of an antenatal acupuncture intervention as an adjunct therapy for antenatal depression (AcuAnteDep): study protocol for a pragmatic randomised controlled trial
Source: Trials. 2016 Feb 17;17:93. doi: 10.1186/s13063-016-1204-9 (PMC4758005; doi:10.1186/s13063-016-1204-9)
Supplement: Additional file 2: — Consent form. (PDF 204 kb) [file 13063_2016_1204_MOESM2_ESM.pdf]

Consent Form - *Adult providing own consent*

**Title**	Acupuncture for Antenatal Depression
**Short Title**	AcuAnteDep
**Protocol Number**	1.0
**Project Sponsor**	University of Western Sydney
**Coordinating Principal Investigator/ Principal Investigator**	Simone Ormsby
**Associate Investigator(s)**	Professors' Smith, Dahlen & Hay & Dr Lind
**Location**	Campbelltown & Camden Hospitals

Declaration by Participant

I have read the Participant Information Sheet or someone has read it to me in a language that I understand.

I understand the purposes, procedures and risks of the research described in the project.

I give permission for my doctors, other health professionals, hospitals or laboratories outside this hospital to release information to the principal investigator Ms Ormsby or the associate investigators involved in this study at the University of Western Sydney, in relation to my depression and treatment for the purposes of this project. I understand that such information will remain confidential.

I have had an opportunity to ask questions and I am satisfied with the answers I have received.

I freely agree to participate in this research project as described and understand that I am free to withdraw at any time during the study without affecting my future health care.

I understand that I will be given a signed copy of this document to keep.

Name of Participant (please print) ______________________________

Signature ______________________ Date ______________________

Name of Witness* to
Participant's Signature (please print) ______________________________

Signature ______________________ Date ______________________

* Witness is not to be the investigator, a member of the study team or their delegate. In the event that an interpreter is used, the interpreter may not act as a witness to the consent process. Witness must be 18 years or older.

Declaration by Study Doctor/Senior Researcher†

I have given a verbal explanation of the research project, its procedures and risks and I believe that the participant has understood that explanation.

Name of Study Doctor/
Senior Researcher† (please print) ____

Signature ______________________________ Date ______________________________

† A senior member of the research team must provide the explanation of, and information concerning, the research project.

Note: All parties signing the consent section must date their own signature.

I understand that, if I decide to discontinue the study treatment, I may be asked to attend follow-up visits to allow collection of information regarding my health status. Alternatively, a member of the research team may request my permission to obtain access to my medical records for collection of follow-up information for the purposes of research and analysis.

I consent to the collection of two blood samples by [insert name] and three saliva samples by Ms Ormsby or Ms Patterson to be taken from me and transported to [insert name] for the use, as described in the relevant section of the Participant Information Sheet, for:

☐ This specific research project
☐ Other research that is closely related to this research project
☐ Any future research.

Name of Participant (please print) ____

Signature ______________________________ Date ______________________________

Name of Witness* to
Participant's Signature (please print) ____

Signature ______________________________ Date ______________________________

* Witness is <u>not</u> to be the investigator, a member of the study team or their delegate. In the event that an interpreter is used, the interpreter may <u>not</u> act as a witness to the consent process. Witness must be 18 years or older.

Name of Study Doctor/
Senior Researcher† (please print) ____

Signature ______________________________ Date ______________________________

† A senior member of the research team must provide the explanation of and information concerning the research project.

Note: All parties signing the consent section must date their own signature.

The conduct of this study at **Campbelltown & Camden Hospitals** has been authorised by the South Western Sydney Local Health District, any person with concerns or complaints about the conduct of this study may also contact the Research Governance Officer on (02) 8738 8304, email: research.support@sswahs.nsw.gov.au and quote project number HREC/14/LPOOL/400 – SSA/14/228.
